# Quality control in cochlear implant therapy: clinical practice guidelines and registries in European countries

**DOI:** 10.1007/s00405-022-07263-4

**Published:** 2022-01-18

**Authors:** A. Loth, C. Vazzana, M. Leinung, D. Guderian, C. Issing, U. Baumann, T. Stöver

**Affiliations:** Department of Oto-Rhino-Laryngology, Head and Neck Surgery, University Hospital Frankfurt, Goethe University, Theodor-Stern-Kai 7, 60590 Frankfurt am Main, Germany

**Keywords:** Cochlear implant, Clinical practice guidelines, Registry, Europe

## Abstract

**Purpose:**

The treatment with a cochlear implant (CI) is the gold standard in therapy of patients with profound hearing loss or deafness. Successful hearing rehabilitation with a CI is a complex, multi-stage process. In medicine, “Clinical Practice Guidelines” (CPG) are widely accepted for the standardization of such processes. These are supplemented by medical registries in which data regarding the treatment can be collected and evaluated. The aim of this paper is to identify currently existing CI-related CPGs and registries in Europe.

**Methods:**

Between 01/2021 and 06/2021, 42 countries on the European continent, including the United Kingdom, Russia and Turkey, were screened using an internet search (search engine: Google) and a key word search in the Pubmed database. Search terms were the respective country name combined with the following terms: “Cochlear Implant”, “CI”, “Cochlear implant clinical practice guideline”, “CI Guideline”, “Cochlear Implant Registry”, “CI Registry”, “Ear nose throat society”. The internet search was conducted in English as well as in the corresponding national language. The objective was to identify a CI-related CPG or registry.

**Results:**

A CPG was found in 16 of 42 (38%) countries. In terms of population, this accounts for 645 million out of 838 million people (77%). A registry existed in 4 of the 42 (10%) countries assessed. This corresponds to 102 million out of 838 million (12%) people. In total, 4 out of 42 countries (10%) had both a CPG and a registry.

**Conclusion:**

Our work shows numerous efforts in Europe to standardize CI care at the national level. While most people in Europe already live in countries with a CPG, this is not the case for CI registries. European-wide consensus on CPGs or registries does not yet exist. The present study thus provides a first assessment of the distribution of CI-related CPGs and registries.

## Introduction

The treatment with a cochlear implant (CI) is the gold standard in therapy of patients with profound hearing loss or deafness. In addition, the medical indication for this treatment has continuously been broadened. Among others, these include bimodal as well as bilateral treatment, the implantation of patients with residual hearing through electric–acoustic stimulation, the treatment of unilaterally deafened patients and the implantation of very old or extremely young people, e.g., children in their first year of life [1–3].

The successful hearing rehabilitation of patients receiving CIs is a multi-stage process consisting of a large number of necessary individual steps. These include audiological evaluation, surgery, fitting of the audio processor, hearing training (rehabilitation) and lifelong follow-up of the implanted patient [4].

Based on information provided by CI manufacturers approved in the USA, the FDA estimates that approximately 737,000 CIs will already have been implanted worldwide by the end of 2019. Even if this does not make it one of the most common interventions worldwide, the calculation with approx. $30,000 US per implant results in a total of approx. $20 billion US in implant costs alone [5]. In addition, there are costs for hearing therapy, speech processor adjustments and consumables.

Consequently, CI treatment is a typical example of a cost-intensive, complex and interdisciplinary medical treatment, from which the necessity of standardized procedures for quality assurance arises.

Such a standardization of treatment procedures has resulted in the creation of so-called “clinical practice guidelines” (CPG) in many areas of patient treatment [6–8]. These CPGs define the currently valid (scientifically based) medical standards of a therapy. For CI care, the “Technology appraisal guidance: Cochlear implants for children and adults with severe to profound deafness” from Great Britain can be pointed out as an illustrative example, which was developed more than 10 years ago for this country [9]. In addition to the introduction of CPGs, the establishment of medical registries is an important aspect of quality assurance in medical treatment. With the help of registries, data on the number of operations, as well as complications and treatment results, for example, can be collected prospectively. A notable example in the field of CI care is the registry of the Swiss Society for Oto-Rhino-Laryngology, Neck- and Facial Surgery, which was established more than 20 years ago [10].

In general, CPGs and registries are developed by the respective medical societies of a country and are used exclusively for the country in question, in the sense of a “national CPG”. As these projects are implemented on a national basis, there are countries in Europe where a CPG and/or a registry exists, as well as countries where these quality assurance tools are not yet in use. These differences between countries located in close proximity are surprising.

To our knowledge so far, there are no data available that has examined the existence of a CPG or a registry for CI care in different countries. Therefore, the aim of the presented study is to survey the existing CPGs and registries for CI care in Europe. To achieve this, the investigation was carried out for the 42 countries of the European continent, including the United Kingdom, Turkey and Russia. The data collected thus represent a valuable preliminary assessment of the currently existing CI treatment standards and registries in Europe.

## Materials and methods

This study did not use patient-related data. Therefore, the vote of an ethics committee was not required.

Forty-two countries on the European continent, including the United Kingdom, Russia and Turkey, were examined in this study (Table 1). The target parameter assessed was the presence of a clinical practice guideline and a registry for cochlear implant therapy in the country in question. A reviewed document was considered a CPG in this survey if the publishing authors classified it as such. There was no need for the CPG to apply to the entire country to which it was allocated.

**Table 1:** Clinical practice guidelines and registries on cochlear implant care in Europe

Number	Country	Population	Guideline (G)/Registry (R)	
1	Albania	2,875,000	–	–
2	Austria	9,041,000	–	–
3	Belgium	11,623,000	G	–
4	Bosnia	3,367,000	–	–
5	Bulgaria	9,613,000	–	–
6	Byelorussia	9,447,000	–	–
7	Croatia	4,088,000	–	–
8	Cyprus	1,213,000	–	–
9	Czech Republic	10,722,000	–	–
10	Denmark	5,805,000	G	–
11	Estonia	1,327,000	–	–
12	Finland	5,546,000	–	–
13	France	65,370,000	G	R
14	Germany	83,964,000	G	–
15	Great Britain	68,127,000	G	–
16	Greece	10,389,000	–	–
17	Hungary	9,643,000	–	–
18	Iceland	342,000	–	–
19	Ireland	4,975,000	G*	–
20	Italy	60,402,000	G	–
21	Kosovo	1,768,000	–	–
22	Latvia	1,872,000	–	–
23	Lithuania	2,697,000	–	–
24	Luxembourg	632,000	–	–
25	Macedonia	2,083,000	–	–
26	Malta	442,000	–	–
27	Moldova	4,027,000	–	–
28	Montenegro	628,000	–	–
29	Netherlands	17,160,000	G	R
30	Norway	5,450,000	–	–
31	Poland	37,818,000	–	–
32	Portugal	10,176,000	G	–
33	Romania	19,152,000	G	–
34	Russia	145,976,000	G	–
35	Serbia	8,713,000	–	–
36	Slovakia	5,461,000	–	–
37	Slovenia	2,079,000	G*	–
38	Spain	46,766,000	G	–
39	Sweden	10,141,000	G	R^+^
40	Switzerland	8,697,000	G	R
41	Turkey	84,958,000	G	–
42	Ukraine	43,559,000	–	–

*Indicates countries where only one hospital with a CI program could be identified and the CPG was published for this hospital. + Indicates there is a registry for children only

For data collection, a general internet search (search engine: Google [11] and a “key word search” in the “Pubmed” database [12] were conducted between 01 and 06/2021. As search terms, the name of the respective country (Table 1) was combined with the following terms: “Cochlear Implant”, “CI”, “Cochlear implant clinical practice guideline”, “CI Guideline”, “Cochlear Implant Registry”, “CI Registry”, “Ear nose throat society”.

The internet search was conducted both in English and in the language of the corresponding country. For this purpose, the search terms were translated with the help of a translator program (“deepl” [13]). The results of the search were saved digitally and the first 50 results were analyzed further. It was determined whether there was a country-specific record of a CPG and a registry. In addition, the date of creation and the date of updating the CPG and the registry were documented.

To establish a reference to the current population of the country examined, the population was extracted from the “worldmeters” database [14].

The results obtained this way were analyzed comparatively and then presented graphically (mapchart.net [15]; Microsoft Excel 365, Microsoft Redmond, Washington, Vereinigte Staaten).

## Results

### Countries with clinical practice guideline

**Fig. 1:**
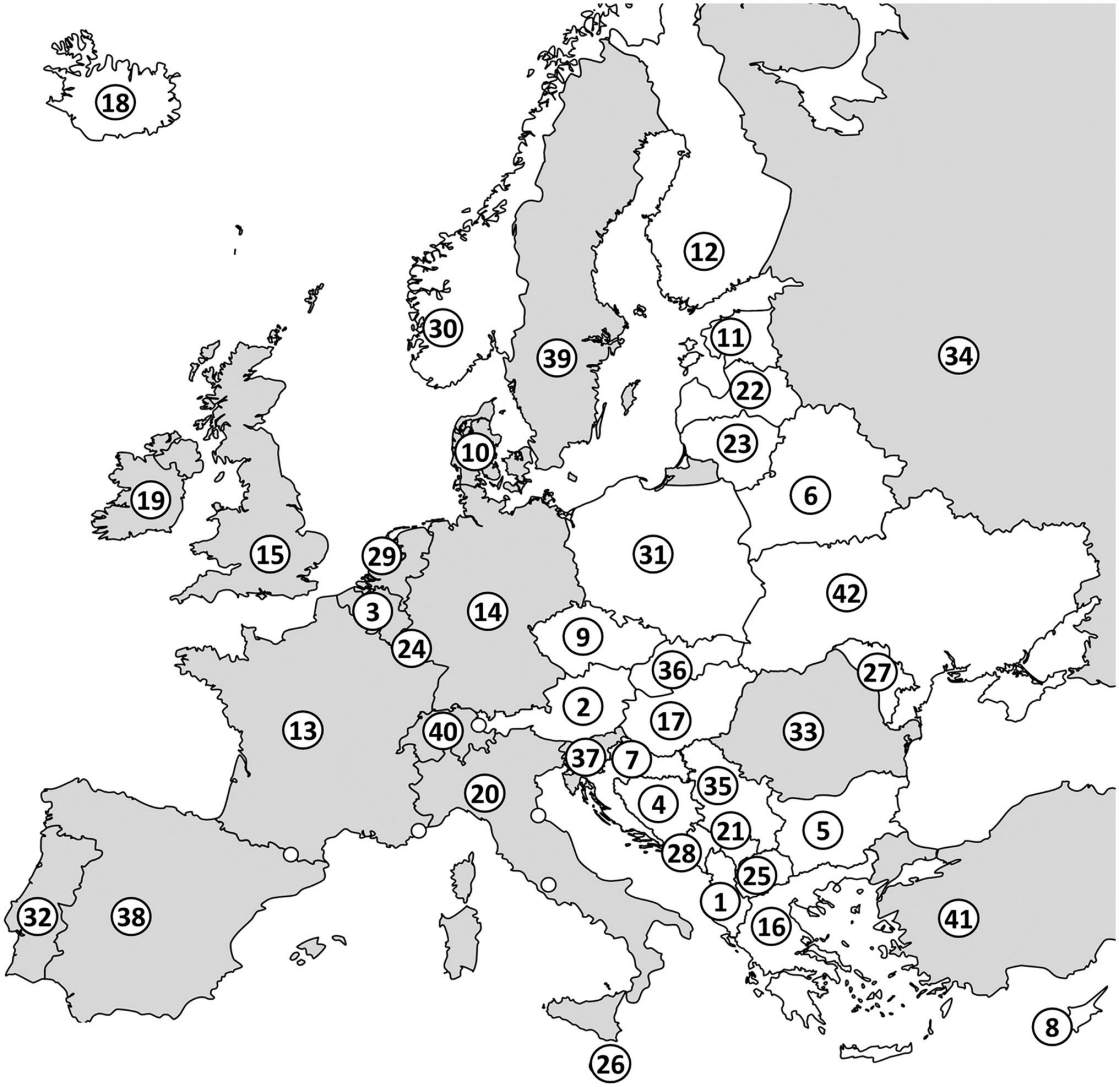
Countries analyzed on the European continent including Turkey and Russia. The numbering of the countries corresponds to the numbers in Table 1. Countries where a cochlear implant guideline or guideline-like format has been identified are shown in gray

Forty-two countries of the European continent, including the Great Britain, Russia and Turkey were screened. By combining the country name with the keywords described above, 1134 internet searches were conducted. A CPG was identified in 16 of the 42 (38%) countries assessed (Fig. 1). In terms of population, a total of 838 million people live in the 42 countries studied. The 16 European countries with a CPG, have a population of 645 million people. This corresponds to 77% (645 out of 838 million).

### Countries with registry

**Fig. 2:**
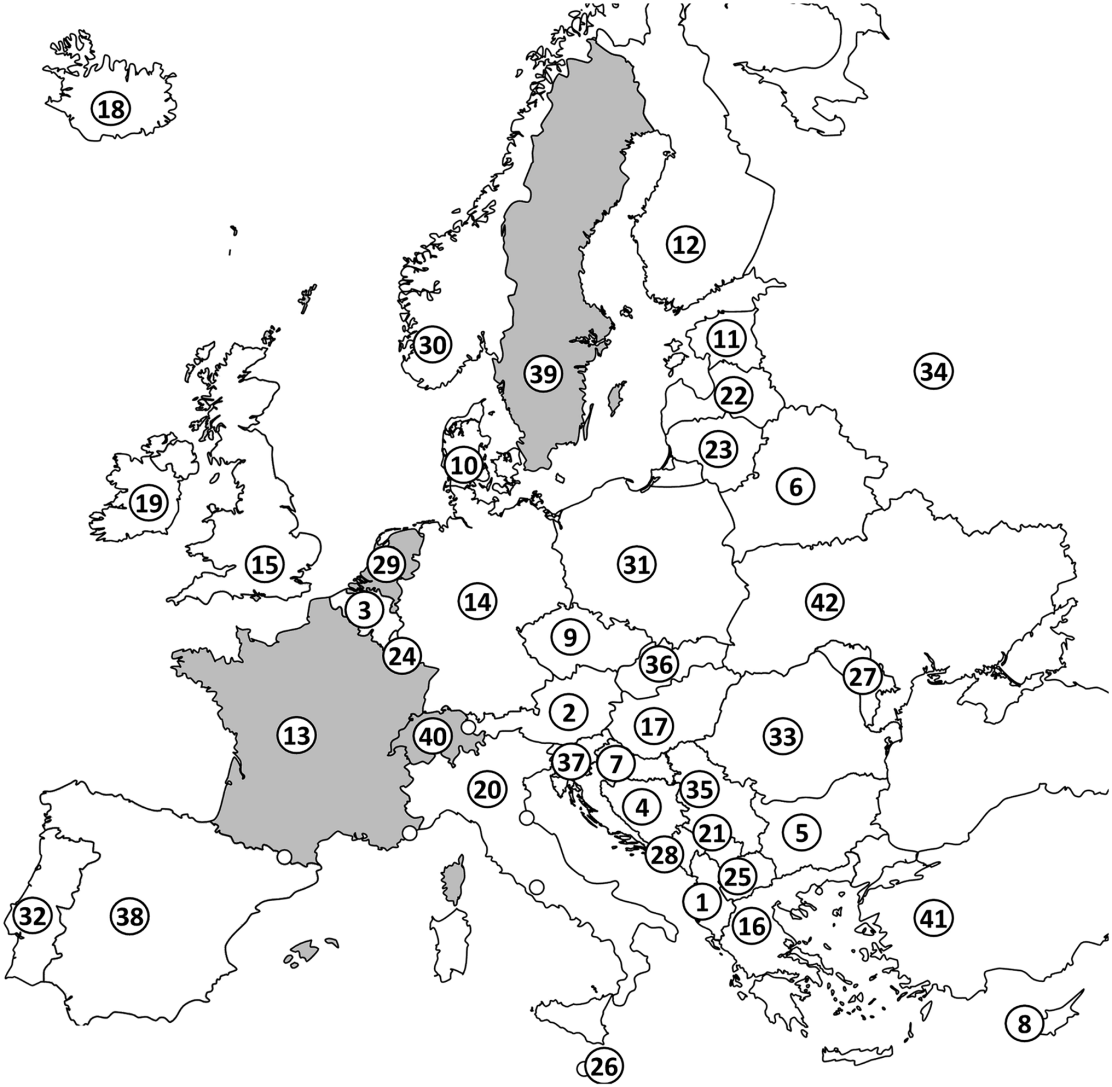
Countries surveyed in continental Europe, including Turkey and Russia. The numbering of the countries corresponds to the numbers in Table 1. Countries where a cochlear implant registry has been identified are shown in gray

Evidence of a registry was found in 4 of the 42 countries analyzed (10%) (Fig. 2). In these four countries, the population was 102 million. This represents 12% (102 out of 838 million) of the people living in the European countries assessed.

### Countries with clinical practice guideline and registry

In 4 out of 42 countries (10%), both a CPG and a registry were found. These countries were Switzerland, Sweden, the Netherlands and France.

### Time of introduction of CPGs

**Fig. 3:**
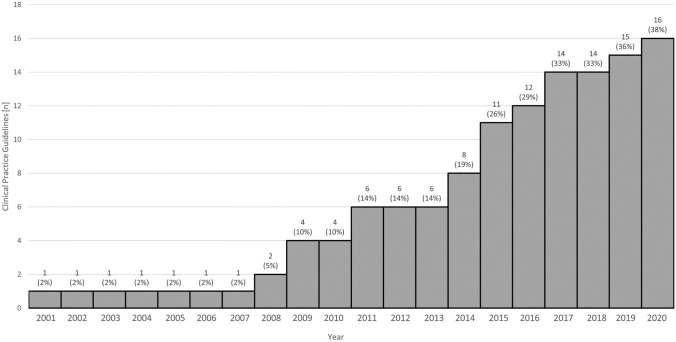
Development over time of guidelines for cochlear implant care in Europe

The identified guidelines were established between 2001 and 2020. Figure 3 shows the date and the development of the number of CPGs in the countries reviewed. While only 8 out of 42 countries (19%) had evidence of a CPG by 2014, this number increased to 16 (38%) in the subsequent period from 2014 to 2020.

## Discussion

Today, hearing rehabilitation with a cochlear implant is the gold standard in the care of people with profound hearing loss or deafness. The process of care is a complex and lifelong measure that demands the highest standards of quality control [4]. In medicine, clinical practice guidelines (CPGs) and registries have become effective tools for quality assurance [4, 7, 16]. With their help, structural requirements and operational processes can be standardized and treatment results as well as complications can be recorded. To our best knowledge, there are currently no studies that have examined the existence of national CPGs and registries in Europe for cochlear implant therapy. This paper presents, for the first time, an overview of the currently established national CI CPGs and CI registries in Europe.

The results show that more than a third (38%) of the countries on the European continent have a CI CPG. In terms of the population of Europe, close to 80% of people live in countries in Europe where a CPG already exists. This difference is surprising but can be explained by the presence of CPGs in very high-population countries. The most populous country, Russia, alone accounts for 17.5% of Europe's inhabitants, with about 146 million people.

The analysis of the countries that do not yet have CPGs shows that they are located particularly in the eastern and south-eastern regions of Europe. The data collected do not provide any clear information on the causes of these regional differences, so that only conjecture is allowed here. Possible explanations could be that the spread of CI treatment differs regionally. In countries where this method is used less frequently, the perceived necessity or acceptance of a CPG could possibly be less pronounced. In addition, the creation of a CPG also requires the initiative of individual stakeholders to take on the task. Therefore, in a country where fewer CI treatments are performed, there may be fewer doctors available to take on the task of creating a CPG.

In conclusion, the reasons for the lack of a CPG remain unclear and should be left to future research.

Noteworthy is the consideration of the time of publication of a CPG in the respective countries. Since 2007, the number of countries with a CPG has increased almost in linear fashion. This observation shows that the need for quality assurance of CI care has been identified in many countries and that attempts have been made to actively shape this process.

The quality of the content of a CPG can vary considerably. The length of the CPGs reviewed clearly demonstrates this, varying from a minimum of 12 pages to a maximum of 32 pages. One reason for this may be that the term CPG is not harmonized internationally. The result is that despite international approaches to standardizing CPGs, such as the “Agree” working group's action guide, the scope, structure and content of CPGs have not been uniformly defined to date [6, 7]. Comparing of the content of different CPGs is also very difficult, as there is no internationally uniform assessment basis for individual steps of CI care. Although international consensus papers have been published in recent years [3, 17], they do not by far cover all possible indications for CI treatment. Therefore, an evaluation of the content could currently only be done in a comparative, but not in an evaluative way. The fact that the national CPGs are usually written in the respective national language also makes comparability difficult. Furthermore, when comparing CPGs a distinction between CPG for CI, care in children and adults must be made. It must therefore be emphasized that no evaluation of the content of the identified CPGs was carried out within this study. Therefore, no statement can be made about the extent to which the identified CPGs comply with the relevant international recommendations.

Objective proof of the benefit of a CPG is very demanding. This is especially true since having CPGs does not necessarily equate to a high quality of care. Although a central institution, usually the respective professional society of a country, produces recommendations, it is not guaranteed that these recommendations are implemented in every case. Here, the use of registries that prospectively collect data on the quality of care can be very helpful. This would allow evidence to be collected on the increased quality of CI care as a result of the use of a CPG, to objectify positive effects and continuously improve the quality of treatment.

To collect these quality indicators of CI care on a larger scale, registries on a national or international level are a good option [15]. According to the data of our study, 10% (4 out of 42) of the countries surveyed now have a registry. This is significantly lower compared to the development in CPGs. Also, in terms of population, only 12% (102 out of 838 million) of the inhabitants live in countries where a registry is maintained. The reasons for these differences were not investigated in the context of this study. However, it is obvious that several important factors could come into consideration. First, the establishment and also the continuous maintenance of a registry are a considerable amount of work that requires financial, technical and human resources. Likewise, the operation of a registry usually requires the support of an organization (e.g., the respective national ENT society), which coordinates operation. Further obstacles could also lie in a lack of willingness or acceptance to participate in a registry. In addition to the factors mentioned, a multitude of other causes could negatively influence the establishment of a registry in a country.

However, the benefit of registries for the further development of therapy is obvious and has already been impressively demonstrated by various large registries, such as the “Trauma Registry”. In this registry, national and international treatment data from 650 hospitals from more than 20 nations are collected. As a result, more than 400,000 treatment cases of trauma patients are now available, which form the basis for a continuous improvement in the quality of care [18]. This example of international cooperation could also serve as a possible blueprint for a future European CI registry.

An example of a successful establishment of a registry is Switzerland. Here, a CI registry has existed since 1992 in which CI data from all five implanting CI centers in Switzerland are recorded [9]. Another remarkable aspect is the fact that data on all implantations performed prior to the establishment of the registry in the early 1990s have also been integrated and are available for analysis [9]. The registry is guided by the Swiss ORL Society and, according to its own information, collects a “minimal dataset” which is obtained from the implanting institutions [9]. An extract of these data is made available to the public at regular intervals in a registry report. In addition, the institutions involved can use the collected data for research purposes [9].

It is noteworthy that even in countries with a high number of CI treatments, a national CI registry does not automatically exist. Germany can be considered as an example in this regard. Although at least 70 hospitals in Germany provide CI care and a CI CPG has already existed since 2001, the national CI registry is currently only in the implementation phase [19, 20]. Other countries where there are currently efforts to establish a CI registry are Great Britain and Belgium. Although in many European countries the clinical implementation of CI care has been very successful in the last decades, there is a relevant potential for improvement with regard to the establishment of national CPGs, and even more so national CI registries. In the long term, the development of a pan-European CPG and registry could also be an interesting perspective for pan-European quality assurance of CI care.

This study has several limitations. One shortcoming is that translation programs had to be used for most of the languages used. Only English, German, French, Italian, Luxembourgish and Greek were translated by native speakers of these languages. It is therefore possible that individual CPGs or registries were not correctly identified due to a language barrier. However, since numerous different queries were searched with different words and word combinations in the respective translated national language, this error does not seem likely. The high number of identified CPGs from countries whose language could only be translated by a translation program (11 out of 16) shows that the method we used was successful.

Another limitation of the study is that only an internet-based search was conducted. CPGs or registries that are not accessible on the internet may not have been identified. While there are certainly countries where CPGs exist which are not publicly accessible, it seems likely that the majority of CPGs and registries were published online, as this allows easy access for practitioners and patients as the target group of the documents. Future research may need to survey the individual national professional societies. This could be done by reaching out to the ORL Societies, the leading experts in the field of CI care or the ministry of health of a respective country and gather the needed information via a questionnaire for example.

In summary, our study demonstrates that there are many efforts in Europe to standardize CI care at the national level. While most people already live in countries where a CPG exists, CI registries are not yet widespread in a comparable way. European-wide consensus on CPGs or registries does not yet exist. The present study thus provides a first assessment of the distribution of CI-related CPGs and registries in Europe and may serve to initiate further registry and CPG programs across Europe.
